# Association between the *CYP1A1 MspI* polymorphism and risk of head and neck cancer: a meta-analysis

**DOI:** 10.1038/s41598-022-05274-z

**Published:** 2022-01-27

**Authors:** Hady Mohammadi, Mehrnoush Momeni Roochi, Farzad Rezaei, Ata Garajei, Hosein Heidar, Bayazid Ghaderi, Masoud Sadeghi

**Affiliations:** 1 Department of Oral and Maxillofacial Surgery, Fellowship in Maxillofacial Trauma, Health Services, Kurdistan University of Medical Sciences, Sanandaj, 6617713446 Iran; 2 Department of Oral and Maxillofacial Surgery, Fellowship in Maxillofacial Trauma, School of Dentistry, Tehran University of Medical Sciences, Tehran, 1439955991 Iran; 3 Department of Oral and Maxillofacial Surgery, School of Dentistry, Kermanshah University of Medical Sciences, Kermanshah, 6713954658 Iran; 4 Department of Head and Neck Surgical Oncology and Reconstructive Surgery, The Cancer Institute, Scholl of Medicine, Tehran University of Medical Sciences, Tehran, 1439955991 Iran; 5 Department of Internal Medicine, Cancer and Immunology Research Center, School of Medicine, Kurdistan University of Medical Sciences, Sanandaj, 6617913446 Iran; 6 Department of Biology, Science and Research Branch, Islamic Azad University, Tehran, 1477893855 Iran

**Keywords:** Genetics, Molecular biology

## Abstract

The studies recommended the relationship between lots of polymorphisms with the head and neck cancers (HNCs) risk. Herein, we reported the association between the *CYP1A1 MspI* polymorphism and the risk of HNC in an updated meta-analysis. The PubMed/MEDLINE, Web of Science, Cochrane Library, and Scopus databases were searched until March 31, 2021, without any restrictions. Odds ratios (ORs) and 95% confidence intervals (CIs) were applied to assess a relationship between *CYP1A1 MspI* polymorphism and the HNC risk based on five applied genetic models by RevMan 5.3 software. Other analyses (sensitivity analysis, meta-regression, and bias analysis) were performed by CMA 2.0 software. Trial sequential analysis (TSA) was done by TSA software (version 0.9.5.10 beta). Among the databases and other sources, 501 recorded were identified that at last, 29 studies were obtained for the analysis. The pooled ORs were 1.28 (95%CI 1.09, 1.51; *P* = 0.003), 1.68 (95%CI 1.16, 2.45; *P* = 0.007), 1.24 (95%CI 1.03, 1.50; *P* = 0.02), 1.26 (95%CI 1.07, 1.48; *P* = 0.005), and 1.66 (95%CI 1.27, 2.16; *P* = 0.0002) for allelic, homozygous, heterozygous, recessive, and dominant models, respectively. Therefore, the m2 allele and m1/m2 and m2/m2 genotypes had significantly increased risks in HNC patients. With regards to stable results and enough samples, the findings of the present meta-analysis recommended that there was an association between *CYP1A1 MspI* polymorphism and the HNC risk.

## Introduction

Head and neck cancer (HNC) affects more than 650,000 cases and 330,000 deaths each year^1^ and has remained a significant public health burden worldwide^2^. Men are significantly more affected by this type of cancer than women with a ratio of 2: 1 to 4: 1 and the prevalence of important anatomical sites of HNC (oral cavity, pharynx, and larynx) varies in different parts of the world^3,4^. HNC's current and future estimated load is shifting to less developed areas that may not have the equipment to cope with this increased load and this requires immediate attention by policymakers through the implementation of effective cancer control policies with population-based interventions^2^. There are many factors that can increase the incidence or prevalence of HNC, including the relative distribution of major risk factors such as alcohol consumption, tobacco, and smoking^5^. Genetic elements have also been implicated in the pathogenesis of this cancer. In support of this statement, several recent meta-analyses have confirmed the relationship of various polymorphisms with the risk of HNC^6–11^. Two reviews confirmed the relationship between several polymorphisms with the risk of HNC^12,13^. Therefore, HNCs are a complex multifactorial disorder that includes genetic, lifestyle, and environmental factors^14,15^. Cytochrome P450 (CYP) enzymes perform a major role in the metabolic activation of polycyclic aromatic hydrocarbons (PAHs) to epoxide intermediates, suggesting a link between PAHs, the CYP pathway, and cancer development that cytochrome P450 1A1 (CYP1A1) is believed to be the most important enzyme in this link^16^ and *CYP1A1*, as a drug-metabolizing enzyme, is among the main enzymes imported in the processing of tobacco-related carcinogens^17^. A studied polymorphism in the *CYP1A1* gene (located on chromosome 15, including 9 exons or chromosome 15q22*–*24) has been shown to be related to the cancer risk, known as *CYP1A1 MspI* polymorphism (CYP1A1*2A)^18^ that *CYP1A1 MspI* is a T → C transition placed downstream of exon 7, in 3′ noncoding region^19^. This polymorphism may change the gene expression level or the messenger RNA stability due to highly induced enzymatic activity^20^. Seven meta-analyses checked the relationship between *CYP1A1 MspI* polymorphism and the risk of HNC including two case–control studies^21^, twelve in Asians with oral cancer^22^, seven^23^, thirty-two^24^, twelve including oral cancer^25^, twelve^26^, and twelve^27^. The meta-analysis He et al.^24^, although had more studies than other meta-analyses, focused on several types of cancer at the same time and didn’t provide information on sensitivity analysis, meta-regression, trial sequential analysis (TSA), and publication bias for HNC. In comparison with our study and other meta-analyses, this meta-analysis included thyroid cancer and different sites of head and neck as HNC, apart from that oral cavity, larynx, and pharynx. In comparison with the meta-analysis of He et al.^24^, we excluded studies that did not have a sufficient number of cases in their groups or their control groups had a deviation from Hardy–Weinberg equilibrium (HWE), because reducing the bias across the studies. Therefore, we aimed to evaluate the connection between the polymorphism of *CYP1A1 MspI* and the risk of HNC with twenty-nine studies in a meta-analysis, meta-regression, and TSA.

## Materials and methods

### Study design

This present study was designed by the Preferred Reporting Items for Systematic Reviews and Meta-Analyses (PRISMA) protocols^28^. The PICO (participants of interest, intervention, control, and outcome of interest) question was: Is *CYP1A1 MspI* polymorphism related to the HNC susceptibility comparing the prevalence of its alleles and genotypes in HNC patients in comparison with controls according to five genetic models?

### Data sources and literature search

A systematic search was comprehensively used in PubMed/MEDLINE, Web of Science, Cochrane Library, and Scopus databases until March 31, 2021, without any restrictions. The used search terms were: (“cytochrome P4501A1” or “CYP1A1” or “AHH” or “aryl hydrocarbon hydroxylase”) and (“oral cancer” or “oral carcinoma” or “oral cavity cancer” or “OSCC” or “oral squamous cell carcinoma” or “oral SCC” or “tongue cancer” or “tongue carcinoma” or “mouth neoplasm” or “head and neck cancer” or “head and neck carcinoma” or “HNSCC” or “salivary gland cancer” or “salivary gland tumor” or “laryngeal cancer” or “larynx Cancer” or “nasopharyngeal cancer” or “nasopharynx cancer” or “Nasopharyngeal carcinoma” or “oropharyngeal cancer” or “oropharyngeal carcinoma” or “hypopharyngeal cancer” or “pharyngeal cancer” or “pharynx cancer” or “hypopharynx squamous cell carcinoma” or “hypopharynx SCC” or “larynx squamous cell carcinoma” or “larynx SCC”) and (“variant” or “polymorphism” or “genotype” or “gene” or “allele”). An independent review of titles and abstracts was conducted by two authors (H.M. and M.S.). A lack of consensus was resolved by a conversation with a third author (M.M.R). We manually checked other electronic sources for relevant studies and also the references of all subject-related studies that met the criteria so that no study was missed.

### Criteria

Inclusion criteria were: (1) studies with a case–control design and reporting the association between *CYP1A1 MspI* polymorphism and the HNC susceptibility; (2) HNC was diagnosed by pathological or histological examinations; (3) sufficient data calculating the allele or genotype frequencies of *CYP1A1 MspI* polymorphism; (4) studies without a deviation from HWE in the control group or studies that HWE could not be computed (because there was no the prevalence of all genotypes separately); (5) Studies having 100 or more than 100 cases in both groups (case and control groups). Exclusion criteria were: (1) duplicate publications; (2) meta-analyses, reviews, letters to the editor, book chapters, conference papers, book chapters; (3) studies in the absence of control group; (4) studies reporting other polymorphisms of *CYP1A1*; and (5) studies reporting the CYP1A1 expression; (5) Studies with less than 100 cases in one or two groups; and (6) family-based studies. Among duplicate publications, we selected one with the newest date. An independent review of full-texts was conducted by two reviewers (H.R.M. and M.S.) and the disagreement was resolved by discussion between both reviewers.

### Data extraction

The data of the involved studies were extracted independently by two reviewers (H.M. and M.S.) to retrieve the necessary information. In case of discrepancy between the data of the two reviewers, a new review was performed by other reviewers (M.M.R and F.R).

### Quality assessment

The quality evaluation was performed according to a questionnaire from the Newcastle–Ottawa scale (NOS)^29^. The NOS included a maximum of nine scores for the least risk of bias in three domains: I) selection of study groups (four scores); II) comparability of groups (two scores); and III) ascertainment of exposure (three scores) for case–control studies^30^. Two reviewers (H.M. and M.S.) independently evaluated the quality of the included studies by scoring them according to a set of pre-established criteria and discrepancies were resolved by a short discussion.

### Statistical analysis

Both odds ratio (OR) and 95% confidence interval (CI) were used to evaluate an association between the polymorphism of *CYP1A1 MspI* and the cancer risk. Five applied genetic models for CYP1A1 *MspI* polymorphism were (allelic (m2 vs. m1), homozygous (m2/m2 vs. m1/m1), heterozygous (m1/m2 vs. m1/m1), recessive (m2/m2 + m1/m2 vs. m1/m1), and dominant (m2/m2 vs. m1/m1 + m1/m2) models). To assess heterogeneity, a Chi-square-based Q test and inconsistency index I^2^ were applied^31,32^ that a *P*-value > 0.10 (I^2^ < 50%) presented a lack of heterogeneity and so we used fixed-effects model^33^ and if there was heterogeneity, the pooled results estimated by the random-effects model^34^.

Subgroup analysis is a method of analysis that involves dividing all participating data into smaller subsets based on a common feature and is often used to compare them and to examine the effects of different factors on the results. We divided the initial results based on ethnicity, control source, and tumor type.

Meta-regression is a quantitative method performed in meta-analysis to estimate the effect of moderators on the effect size of the study applying regression-based techniques^35^. We assessed the effect of publication year and sample size on the effect size.

There were two sensitivity analyses containing “one-study-removed” and cumulative analysis” to evaluate the stability/consistency of pooled results.

Funnel plots are visual tools for evaluating the types of biases in meta-analyses and are designed to examine whether publication bias can affect the reliability of estimates^36^. Both Begg’s^37^ and Egger’s^38^ tests were used for the diagnosis of asymmetry of these plots. Asymmetry can be a reason for bias in studies that in the state, *P*-values (two-sided) < 0.05 for the tests.

The *P*-values (two-sided) < 0.05 was as a significant index. The results of forest plot analyses were extracted by Review Manager 5.3 (RevMan 5.3) software and other analyses by Comprehensive Meta-Analysis version 2.0 (CMA 2.0) software.

We used TSA due to false-positive or negative conclusion^39^ in the meta-analysis using TSA software (version 0.9.5.10 beta) (Copenhagen Trial Unit, Centre for Clinical Intervention Research, Rigshospitalet, Copenhagen, Denmark) to reduce these statistical errors^40^. The required information size (RIS) was calculated when an alpha risk of 5%, a beta risk of 20%, and a two-sided boundary type were used. While the Z-curve reached the RIS line or monitoring the boundary line or futility area, it illustrated that enough samples are involved in the studies, and therefore their results were valid. Otherwise, the value of information was not great enough, and additional studies were needed.

### Ethics approval and consent to participate

All methods were performed in accordance with the relevant guidelines and regulations.

## Results

### Study selection

**Figure 1:**
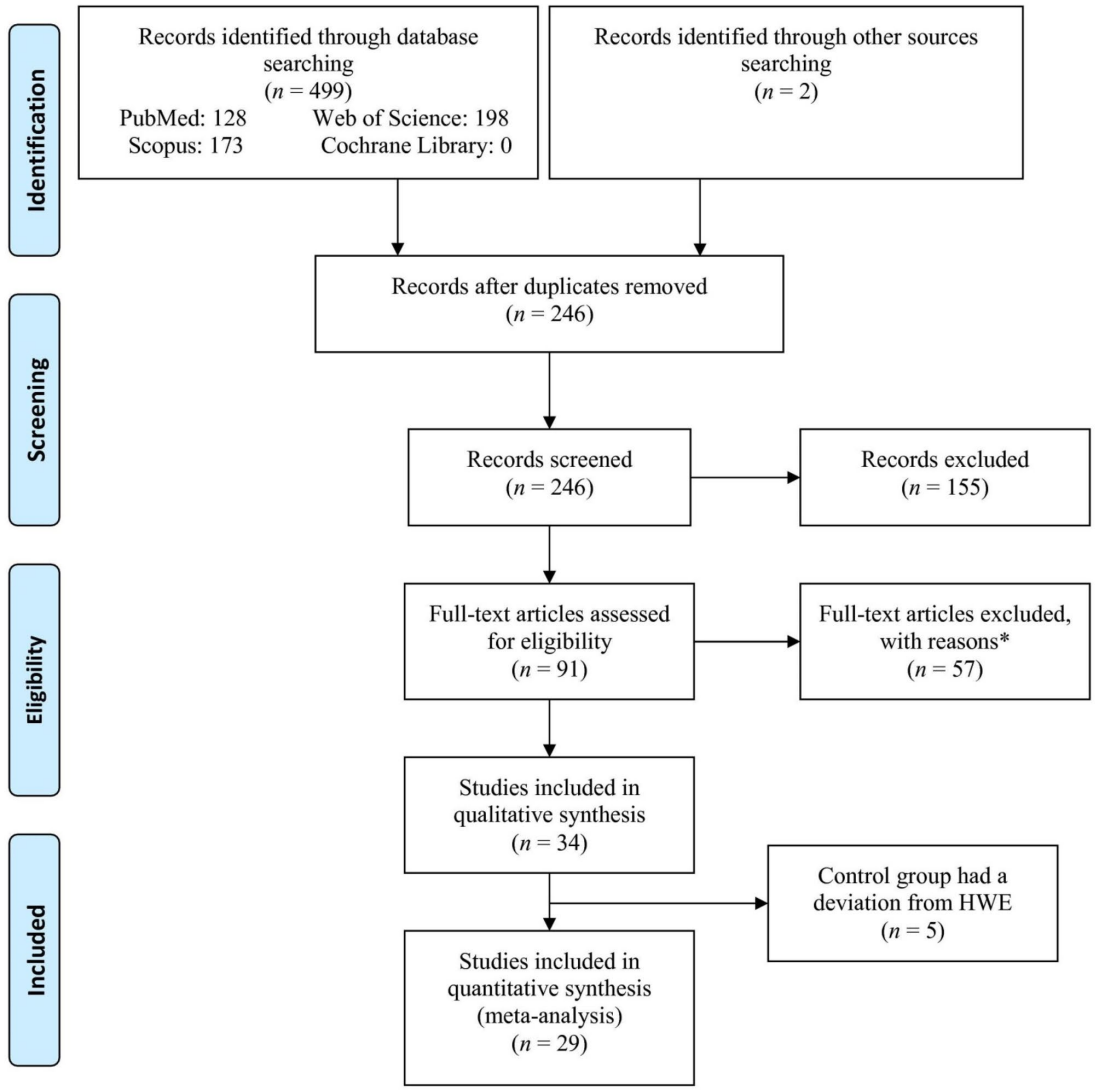
Flowchart of the study selection.

Among the databases and other sources, 501 recorded were identified (Fig. 1). After omitting duplicates and unrelated records, 91 full-text articles were evaluated for eligibility. Then, 57 articles excluded with reasons (one mixed oral precancerous and cancer cases, one had no control group, two reviews, four reported CYP1A1 expression, two didn’t report the prevalence of alleles and genotypes, one book chapter, twenty-two reported other polymorphisms of *CYP1A1*, two reported duplicate publications, one family-based study, one had no sufficient data, one reported oral precancerous cases, twelve studies reported less than 100 cases in one or two groups (case and control groups), and seven meta-analyses). After that, 34 studies^17,41–73^ were included systematic review and we deleted 5 studies^41,53,66,71,72^ with a deviation from HWE in their control groups. Finally, 29 studies were entered into the analysis.

### Basic characteristics

Table 1 is shown the characteristics of the studies^17,42–52,54–65,67–70,73^ involved in the meta-analysis. The studies were published from 1996 to 2019 including 8392 HNC cases and 8646 controls. Eighteen studies^43–46,48,52,55,59,61–66,68–70,73^ were reported in Asians, seven^17,42,49,51,54,57,58^ in Caucasians, and four^47,50,56,60^ in mixed ethnicity. The control source in eighteen studies^42–44,47,49–52,54,58,60–63,66,69,70,73^ was hospital-based and in eleven^17,45,46,48,55–57,59,64,65,68^ was population-based. The type of tumor and the genotyping method were other variables for the studies.

**Table 1:** Basic characteristics of included studies in the meta-analysis.

First author, publication year	Country	Ethnicity	Cases	Controls	Source of controls	Tumor type	Genotyping method
Lucas^57^	France	Caucasian	302	253	PB	Oral, laryngeal, and pharyngeal cancers	PCR–RFLP
Sato^64^	Japan	Asian	142	142	PB	Oral cancer	PCR
Tanimoto^73^	Japan	Asian	100	100	HB	Oral cancer	PCR–RFLP
Ko^54^	Germany	Caucasian	195	177	HB	Oral, laryngeal, and pharyngeal cancers	PCR–RFLP
Cheng^45^	Taiwan	Asian	172	218	PB	Pharyngeal cancer	PCR–RFLP
Gronau^51^	Germany	Caucasian	187	139	HB	Oral, laryngeal, and pharyngeal cancers	PCR-RFLPAS-PCR
Matthias^58^	Germany	Caucasian	335	205	HB	Oral, laryngeal, and pharyngeal cancers	PCR–RFLP
Gajecka^49^	Poland	Caucasian	213	149	HB	Laryngeal cancer	PCR–RFLP
Gattás^50^	Brazil	Mixed	103	102	HB	Oral, laryngeal, and pharyngeal cancers	PCR–RFLP
Boccia^42^	Italy	Caucasian	210	245	HB	Oral, laryngeal, and pharyngeal cancers	PCR–RFLP
Sam^63^	India	Asian	408	220	HB	Oral, laryngeal, and pharyngeal cancers	PCR–RFLP
Singh^68^	India	Asian	200	200	PB	Oral, laryngeal, and pharyngeal cancers	PCR–RFLP
Olivieri^60^	Brazil	Mixed	153	145	HB	Oral, laryngeal, and pharyngeal cancers	PCR–RFLP
Chatterjee^43^	India	Asian	102	100	HB	Oral cancer	PCR
Sabitha^61^	India	Asian	150	145	HB	Oral, laryngeal, and pharyngeal cancers	PCR–RFLP
Sam^62^	India	Asian	408	220	HB	Oral, laryngeal, and pharyngeal cancers	PCR
Sharma^65^	India	Asian	203	201	PB	Oral, laryngeal, and pharyngeal cancers	PCR–RFLP
Lourenço^56^	Brazil	Mixed	142	142	PB	Oral, laryngeal, and pharyngeal cancers	PCR–RFLP
Cury^47^	Brazil	Mixed	313	417	HB	Oral, laryngeal, and pharyngeal cancers	PCR–RFLP
Guo^52^	China	Asian	300	300	HB	Oral cancer	PCR
Shukla^67^	India	Asian	100	100	HB	Oral cancer	PCR–RFLP
Singh^69^	India	Asian	122	127	HB	Oral cancer	PCR–RFLP
Choudhury^46^	India	Asian	180	240	PB	Oral, laryngeal, and pharyngeal cancers	PCR–RFLP
Lourembam^55^	India	Asian	105	115	PB	Pharyngeal cancer	PCR–RFLP
Maurya^59^	India	Asian	750	749	PB	Oral, laryngeal, and pharyngeal cancers	PCR–RFLP
Singh^70^	India	Asian	170	230	HB	Oral, laryngeal, and pharyngeal cancers	PCR–RFLP
Zakiullah^17^	Pakistan	Caucasian	200	151	PB	Pharyngeal cancer	RT-PCR
Dong^48^	China	Asian	750	750	PB	Oral cancer	PCR–RFLP
Chen^44^	China	Asian	874	874	HB	Oral cancer	PCR

### Quality assessment

Ten criteria were identified to evaluate the quality of the studies contained in the meta-analysis (Table 2). Twenty-five studies had a high quality (score ≥ 7).

**Table 2:** Criteria of quality assessment based on Newcastle–Ottawa Scale (NOS).

First author, publication year	Selection	Comparability	Exposer	NOS score
Lucas^57^	****	*	***	8
Sato^64^	****	**	***	9
Tanimoto^73^	**	**	***	7
Ko^54^	***	*	***	7
Cheng^45^	***	**	***	7
Gronau^51^	**	**	***	7
Matthias^58^	**	*	***	6
Gajecka^49^	***	-	***	6
Gattás^50^	**	**	**	6
Boccia^42^	**	**	***	7
Sam^63^	**	**	***	7
Singh^68^	****	**	***	9
Olivieri^60^	****	**	***	9
Chatterjee^43^	****	**	***	9
Sabitha^61^	****	**	***	9
Sam^62^	***	**	***	8
Sharma^65^	****	**	***	9
Lourenço^56^	****	**	***	9
Cury^47^	**	**	***	7
Guo^52^	**	*	***	6
Shukla^67^	***	**	***	8
Singh^69^	***	**	***	8
Choudhury^46^	****	**	***	9
Lourembam^55^	****	**	***	9
Maurya^59^	****	**	***	9
Singh^70^	***	**	***	8
Zakiullah^17^	****	*	***	8
Dong^48^	****	**	***	9
Chen^44^	***	**	***	8

Each asterisk shows one score.

### Genotype prevalence

Table 3 is shown the genotype prevalence of *CYP1A1 MspI* polymorphism in the HNC patients and the controls. Seven studies^17,44,46,48,50,52,62^ had not reported any data about HWE.

**Table 3:** Prevalence of genotypes of *CYP1A1 MspI* polymorphism in the patients with head and neck cancer (cases) and the controls.

First author, publication year	Case			Control			*P*-value of HWE in controls
	m1/m1	m1/m2	m2/m2	m1/m1	m1/m2	m2/m2	
Lucas^57^	235	66	1	212	38	3	0.389
Sato^64^	56	55	31	62	65	15	0.737
Tanimoto^73^	32	53	15	62	30	8	0.126
Ko^54^	158	36	1	146	29	2	0.681
Cheng^45^	74	75	23	83	96	39	0.226
Gronau^51^	142	45	0	113	24	2	0.581
Matthias^58^	290	44	1	184	19	2	0.074
Gajecka^49^	191	21	1	230	18	1	0.325
Gattás^50^	65	38		63	39		NA
Boccia^42^	169	41		189	56		> 0.05
Sam^63^	146	199	63	115	91	14	0.475
Singh^68^	109	75	16	135	56	9	0.312
Olivieri^60^	133	20	0	106	39	0	0.061
Chatterjee^43^	30	46	26	42	39	19	0.077
Sabitha^61^	40	73	37	71	66	8	0.141
Sam^62^	146	262		115	105		NA
Sharma^65^	107	74	22	129	66	6	0.479
Lourenço^56^	90	52		91	51		> 0.05
Cury^47^	207	106		262	155		> 0.05
Guo^52^	185		115	237		63	NA
Shukla^67^	60	30	10	48	46	6	0.241
Singh^69^	60	45	17	50	58	19	0.746
Choudhury^46^	80	100		130	110		NA
Lourembam^55^	27	50	28	28	48	39	0.091
Maurya^59^	391	280	79	451	254	44	0.304
Singh^70^	77	70	23	125	83	22	0.140
Zakiullah^17^	124	76		96	55		NA
Dong^48^	463		287	593		157	NA
Chen^44^	318	556		468	406		NA

*HWE* Hardy–Weinberg equilibrium. *NA * Not available.

### Pooled analyses

**Figure 2:**
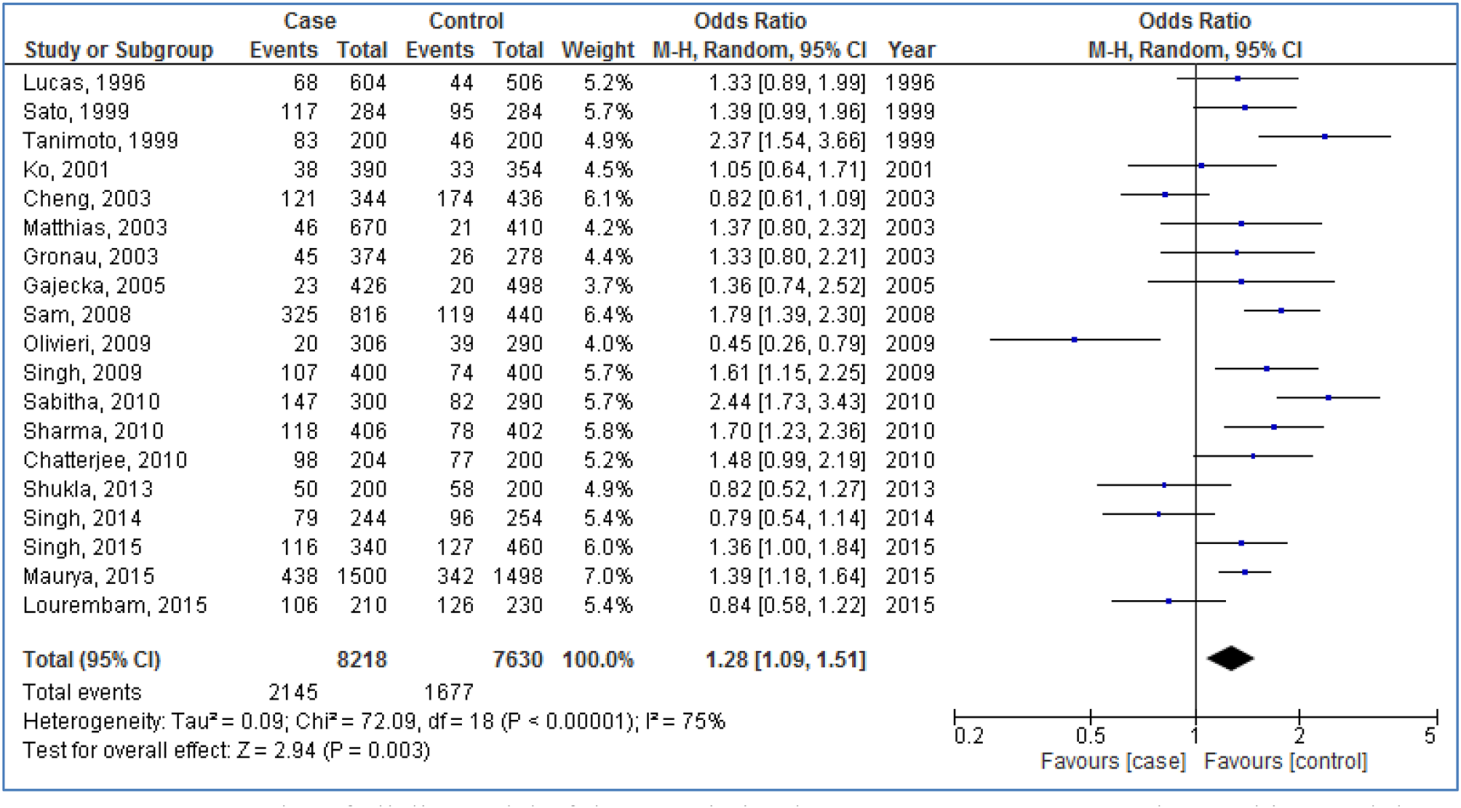
Forest plot of allelic model of the association between *CYP1A1 MspI* polymorphism and the risk of head and neck cancer.

**Figure 3:**
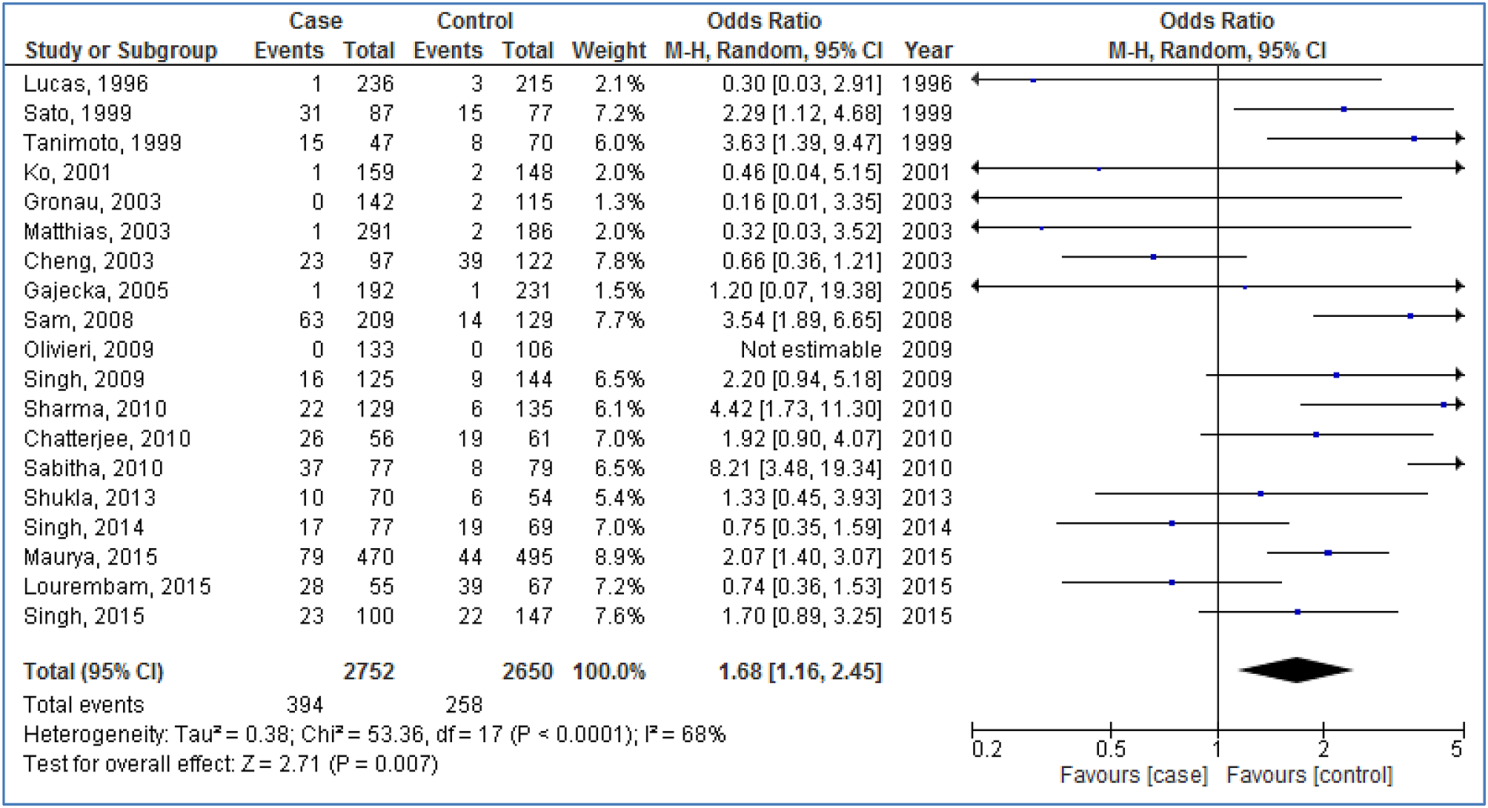
Forest plot of homozygous model of the association between *CYP1A1 MspI* polymorphism and the risk of head and neck cancer.

**Figure 4:**
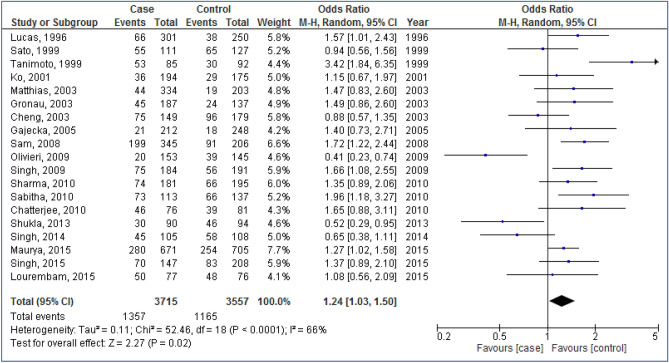
Forest plot of heterozygous model of the association between *CYP1A1 MspI* polymorphism and the risk of head and neck cancer.

**Figure 5:**
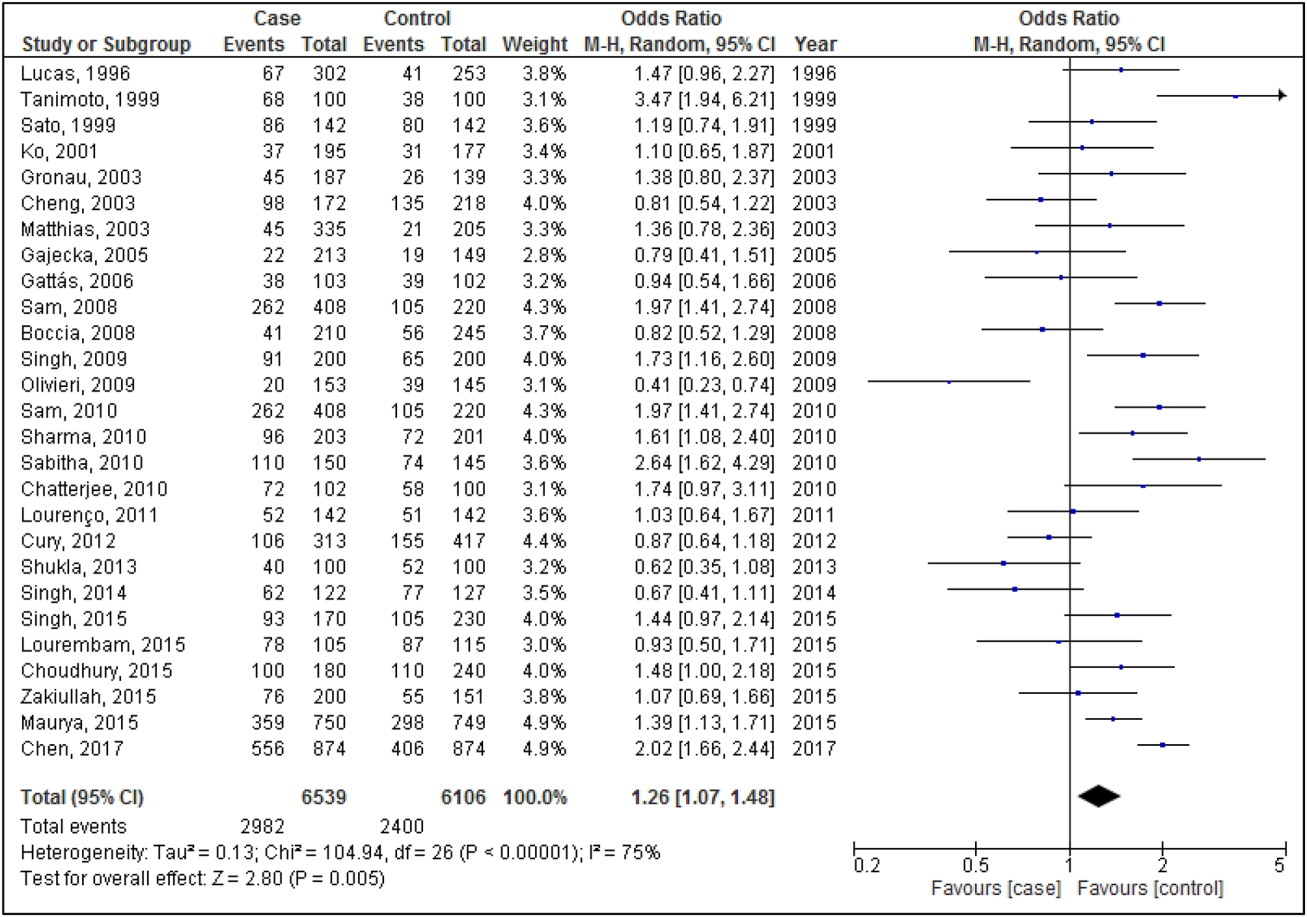
Forest plot of recessive model of the association between *CYP1A1 MspI* polymorphism and the risk of head and neck cancer.

**Figure 6:**
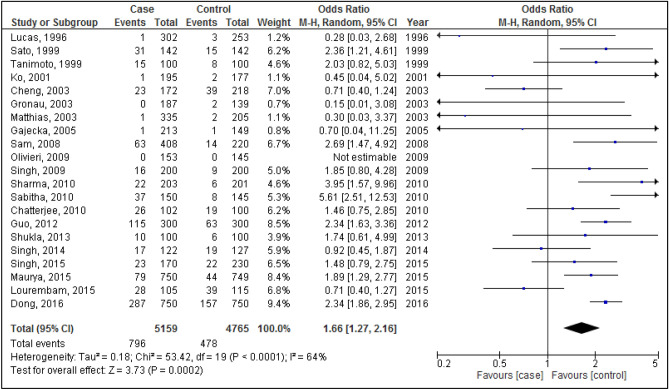
Forest plot of dominant model of the association between *CYP1A1 MspI* polymorphism and the risk of head and neck cancer.

Figures 2, 3, 4, 5 and 6 are shown the random-effects analyses of allelic, homozygous, heterozygous, recessive, and dominant models of the association between *CYP1A1 MspI* polymorphism and the risk of HNC, respectively. The pooled ORs were 1.28 (95%CI 1.09, 1.51; *P* = 0.003; I^2^ = 75%) for allelic, 1.68 (95%CI 1.16, 2.45; *P* = 0.007; I^2^ = 68%) for homozygous, 1.24 (95%CI 1.03, 1.50; *P* = 0.02; I^2^ = 66%) for heterozygous, 1.26 (95%CI 1.07, 1.48; *P* = 0.005; I^2^ = 75%) for recessive, and 1.66 (95%CI 1.27, 2.16; *P* = 0.0002; I^2^ = 64%) for dominant models. The m2 allele and m1/m2 and m2/m2 genotypes had significantly an elevated risk in HNC patients.

### Subgroup analyses

The subgroup analysis was performed on the ethnicity, the control source, and the tumor type (Table 4). The results showed that ethnicity, control source, and tumor type could be effective factors on the pooled ORs. With regard to the ethnicity, the association of *CYP1A1 MspI* polymorphism and HNC risk based on five models (allelic, homozygous, heterozygous, recessive, and dominant), two models (allelic and heterozygous)), and two models (allelic and heterozygous) were statistically significant for Asian, Caucasian, and mixed ethnicities, respectively, that in contrast with Asian and Caucasian ethnicities, there was a decreased risk of m2 allele and m1/m2 genotype in mixed ethnicity. For the control source, the association was statistically significant in four models (allelic, homozygous, and dominant) for hospital-based controls and three models (heterozygous and recessive) for population-based controls. For tumor type, the association in four models (allelic, homozygous, recessive, and dominant) for oral cancer, three models (allelic, homozygous, and dominant) for laryngeal cancer, and three models (allelic, heterozygous, and recessive) for pharyngeal cancer was statistically significant.

**Table 4:** Subgroup analysis of association between the head and neck cancer risk and *CYP1A1 MspI* polymorphism.

Subgroup (N,N’,N’’)	m2 versus m1	m2/m2 versus m1/m1	m1/m2 versus m1/m1	m2/m2 + m1/m2 versus m1/m1	m2/m2 versus m1/m1 + m1/m2
	OR (95%CI), *P*, I^2^	OR (95%CI), *P*, I^2^	OR (95%CI), *P*, I^2^	OR (95%CI), *P*, I^2^	OR (95%CI), *P*, I^2^
All (19,27,21)	**1.28** (1.09, 1.51), 75%	**1.68** (1.16, 2.45), 68%	**1.24** (1.03, 1.50), 66%	**1.26** (1.07, 1.48), 75%	**1.66** (1.27, 2.16), 64%
Ethnicity					
Asian (13,16,15)	**1.35** (1.12, 2.64), 79%	**1.94** (1.32, 2.86), 73%	**1.28** (1.02, 1.59), 67%	**1.47** (1.20, 1.81), 76%	**1.80** (1.39, 2.34), 68%
Caucasian (5,7,5)	**1.28** (1.02, 1.59), 0%	0.37 (0.12, 1.12), 0%	**1.42** (1.12, 1.81), 0%	1.13 (0.94, 1.36), 0%	0.32 (0.11, 0.98), 0%
Mixed (1,4,1)	**0.45** (0.26, 0.79)	Not estimable	**0.41** (0.23, 0.74)	0.79 (0.56, 1.12), 0.19	Not estimable
Source of controls					
Hospital-based (13,17,13)	**1.32** (1.04, 1.66), 77%	**1.99** (1.19, 3.33), 65%	1.25 (0.94, 1.66), 74%	1.25 (1.97, 1.61), 82%	**1.97** (1.60, 2.41), 44%
Population-based (6,10,8)	1.20 (0.96, 1.50), 70%	1.29 (0.74, 2.23), 71%	**1.24** (1.07, 1.45), 25%	**1.30** (1.16, 1.46), 26%	1.51 (0.94, 2.44), 81%
Tumor type*					
Oral cancer (9,12,11)	**1.53** (1.17, 2.00), 78%	**2.03** (1.43, 2.86), 59%	1.10 (0.88, 1.38), 64%	**1.32** (1.05, 1.66), 76%	**2.12** (1.85, 2.43), 75%
Laryngeal cancer (5,7,5)	**1.91** (1.61, 2.26), 48%	**2.65** (1.11, 6.31), 77%	1.33 (0.87, 2.05), 68%	1.44 (0.98, 2.12), 60%	**2.55** (1.75, 3.70), 44%
Pharyngeal cancer (4,6,4)	**1.44** (1.03, 2.00), 80%	2.11 (1.00, 4.44), 79%	**1.38** (1.14, 1.67), 46%	**1.39** (1.04, 1.87), 62%	1.78 (0.97, 3.29), 78%

*Some studies analyzed the data for head and neck cancers separately, too. All models included 19 studies, except for recessive (m2/m2 + m1/m2 vs. m1/m1) and dominant (m2/m2 vs. m1/m1 + m1/m2) models including 27 and 21 studies, respectively. N: number of studies in allelic, homozygous, and heterozygous models. N’: number of studies for recessive model. N’’: number of studies for dominant model. *OR* Odds ratio, *CI* Confidence interval. Bold data means statistically significant (*P* < 0.05).

### Publication bias

**Figure 7:**
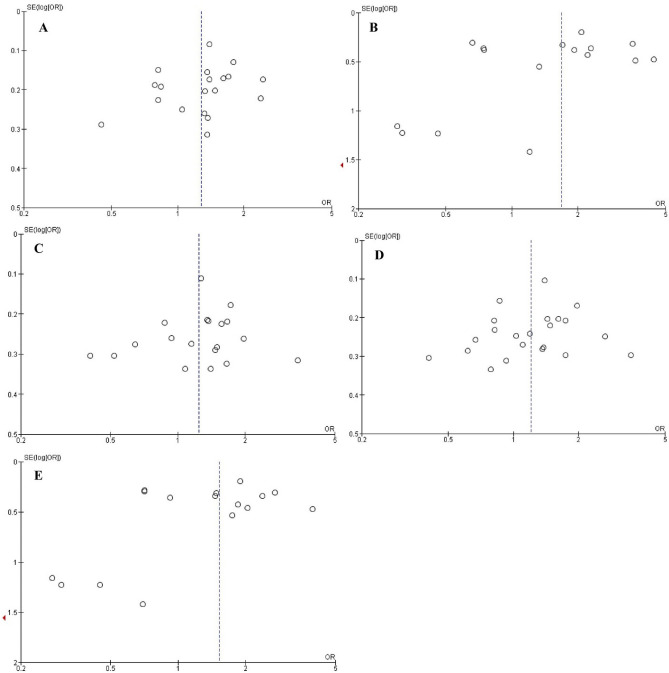
Funnel plots of the association between *CYP1A1 MspI* polymorphism and the risk of head and neck cancer. (**A**) Allelic model; (**B**) Homozygous model; (**C**) Heterozygous model; (**D**) Recessive model; (**E**) Dominant model.

Figure 7 is shown the funnel plots of the relationship between *CYP1A1 MspI* polymorphism and the risk of HNC based on the genetic models. Both Egger’s and Begg’s tests were: (allelic model: 0.322 and 0.151; homozygous model: 0.340 and 0.471; heterozygous model: 0.570 and 0.421; recessive model: 0.030 and 0.050; and dominant model: 0.064 and 0.243). The *P*-values > 0.05 were for both tests that determined lack of any publication bias across the studies, exception for Egger’s test in dominant model (*P* < 0.05) that showed the publication bias across the studies in this model.

### Trial sequential analysis

**Figure 8:**
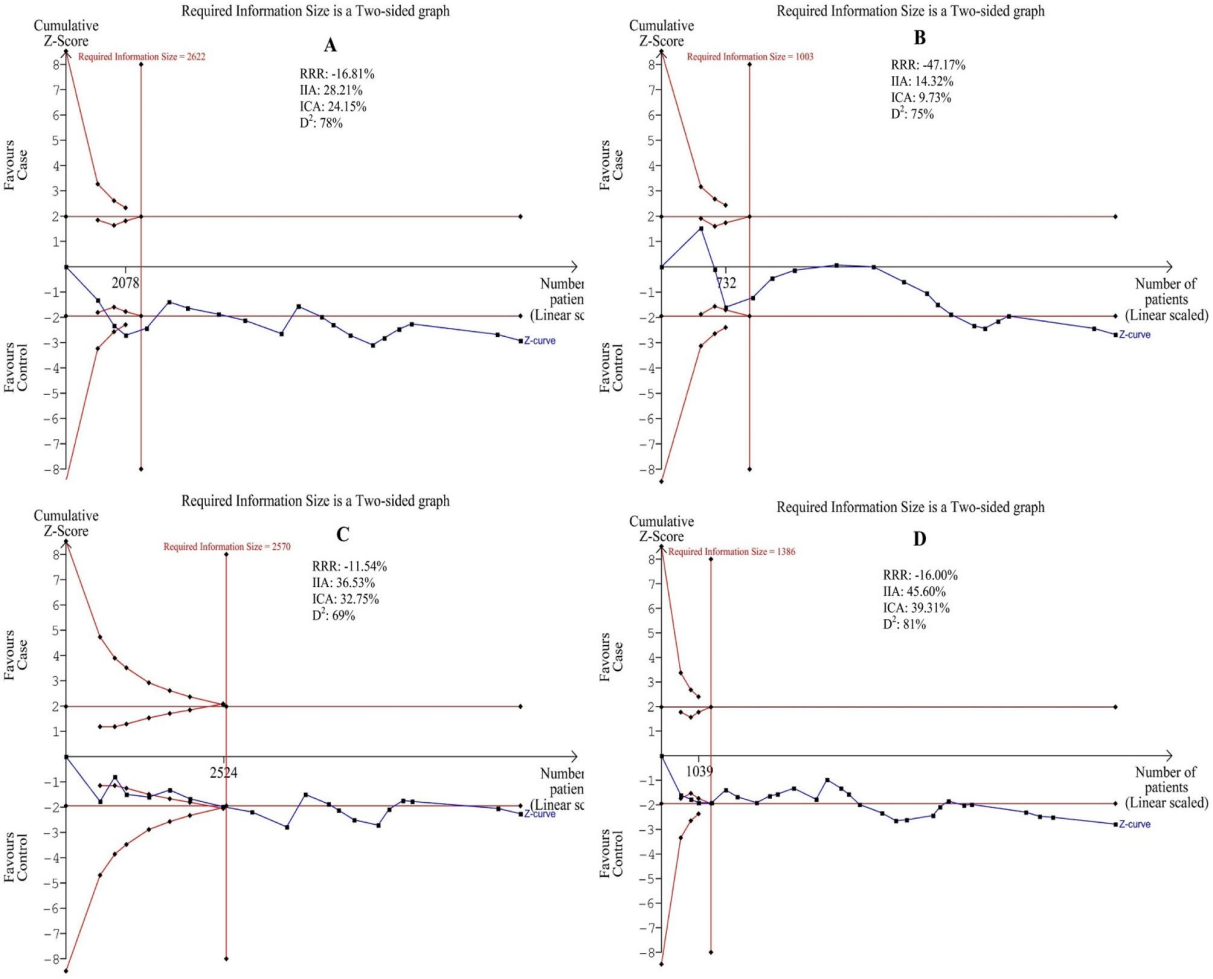
Trial sequential analyses for *CYP1A1 MspI* polymorphism and the head and neck risk. (**A**,**B**,**C**,**D**,**E**) show allelic, homozygous, heterozygous, and recessive models, respectively. Abbreviation: D^2^, diversity; RRR, relative risk reduction; IIA, incidence in intervention arm; ICA, incidence in control arm. IIA and ICA were calculated from the average incidence in case and control groups. Error α and 1 − β were defined as 5% and 80%, respectively in each model.

The Z-curve (blue line) of the allelic, homozygous, heterozygous, recessive, and dominant models reached the RIS line (vertical red line), revealing that the *CYP1A1 MspI* polymorphism was related to the HNC risk with enough samples and reliable results that we selected the graphs for four models because of the better quality of the graphs (Fig. 8).

### Sensitivity analysis

**Figure 9:**
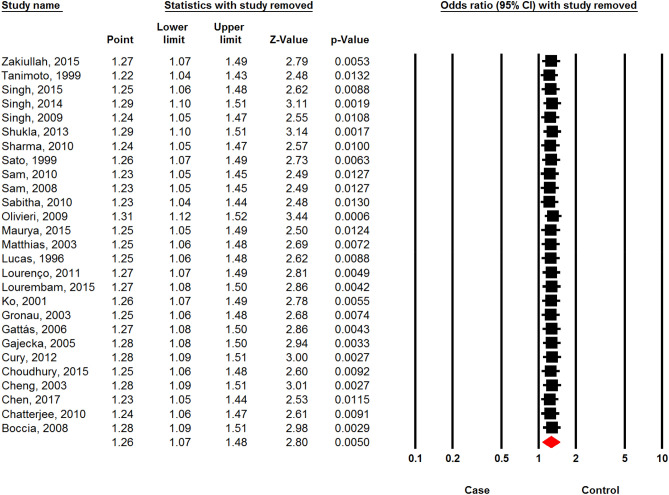
“One-study-removed” analysis of the association between *CYP1A1 MspI* polymorphism and the risk of head and neck cancer based on recessive model.

**Figure 10:**
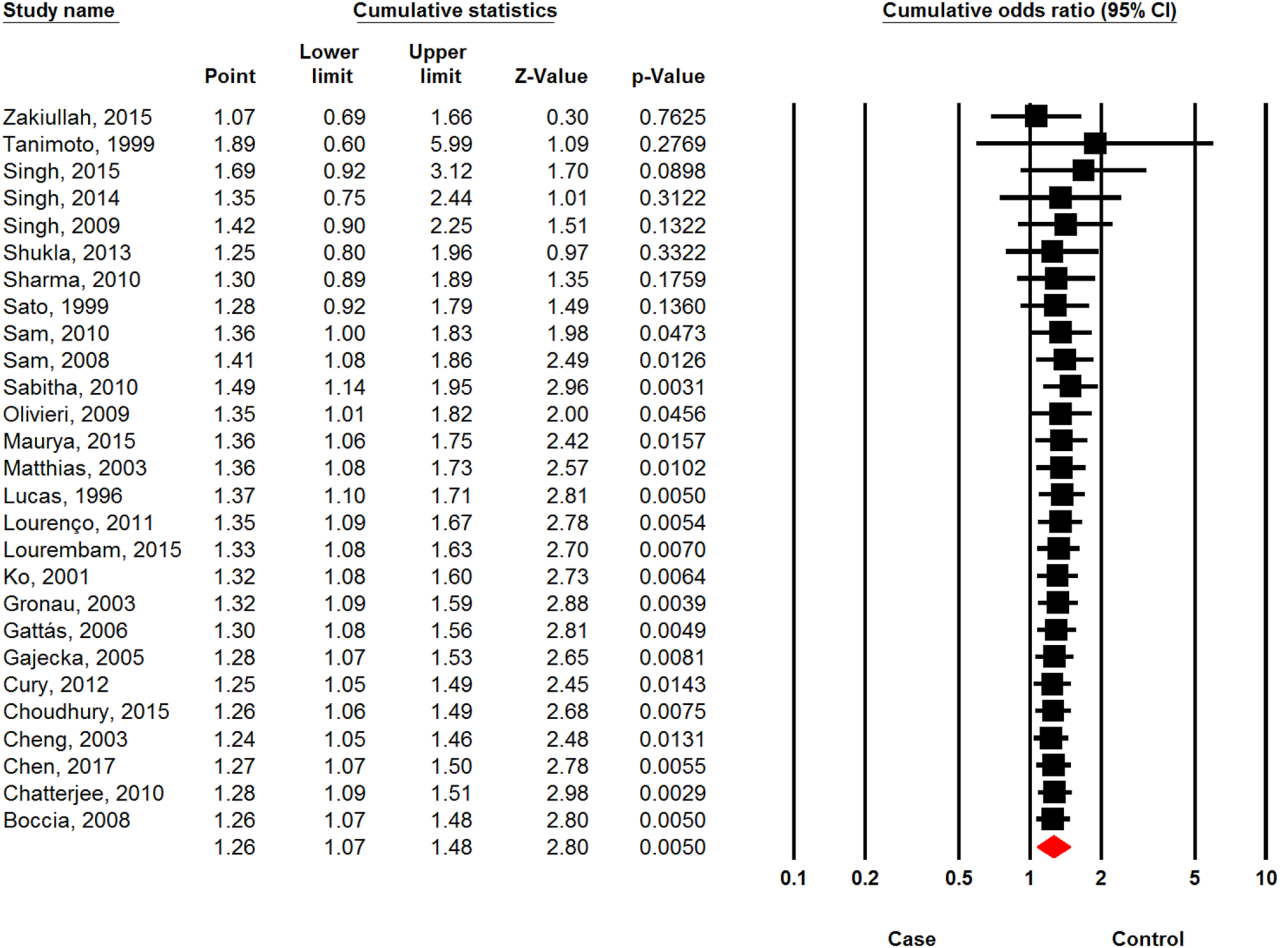
Cumulative analysis of the association between *CYP1A1 MspI* polymorphism and the risk of head and neck cancer based on recessive model.

The sensitivity analyses including “one-study-removed” (Fig. 9) and “cumulative analysis” (Fig. 10) showed the stability of the initial pooled ORs. We included the results of the sensitivity analyses for the recessive model.

### Meta-regression

A meta-regression analysis based on the publication year and the sample size were carried out on the relationship between the HNC risk and *CYP1A1 MspI* polymorphism (Table 5). The analysis showed the sample size in recessive and dominant models, the tumor type in allelic, homozygous, and heterozygous models, and the ethnicity in allelic, homozygous, recessive, and dominant models could be important confounding factors for the association between the HNC risk and *CYP1A1 MspI* polymorphism (*P* < 0.05). Increasing the sample size, the risk of HNC significantly increased (a direct correlation).

**Table 5:** Fixed-effect meta-regression (the slope values) of log odds ratio versus five variables.

Genetic models	Point estimate	95%CI		Z-value	*p*-value
		Lower limit	Upper limit		
**Publication year**					
m2 versus m1	− 0.00446	− 0.01765	0.00874	− 0.66153	0.50827
m2/m2 versus m1/m1	0.00192	− 0.03163	0.03548	0.11234	0.91056
m1/m2 versus m1/m1	− 0.00587	− 24.28170	0.01221	− 0.63615	0.52468
m2/m2 + m1/m2 versus m1/m1	0.01072	− 0.00285	0.02429	1.54865	0.12147
m2/m2 versus m1/m1 + m1/m2	0.02123	− 0.00517	0.04763	1.57607	0.11501
**Sample size**					
m2 versus m1	0.00009	− 0.00007	0.00025	1.14771	0.25109
m2/m2 versus m1/m1	0.00022	− 0.00016	0.00060	1.15662	0.24743
m1/m2 versus m1/m1	0.00008	− 0.00015	0.00030	0.67630	0.49885
m2/m2 + m1/m2 versus m1/m1*	0.00027	0.00013	0.00040	3.86487	0.00011
m2/m2 versus m1/m1 + m1/m2*	0.00032	0.00008	0.00055	2.65233	0.00799
**Tumor type**					
m2 versus m1*	0.09625	0.03383	0.15866	3.02236	0.00251
m2/m2 versus m1/m1*	0.20208	0.06035	0.34380	2.79453	0.00520
m1/m2 versus m1/m1*	0.11909	0.02192	0.21625	2.40207	0.01630
m2/m2 + m1/m2 versus m1/m1	− 0.05306	− 0.11195	0.00584	− 1.76562	0.07746
m2/m2 versus m1/m1 + m1/m2	− 0.01062	− 0.10919	0.08795	− 0.21121	0.83272
**Source of control**					
m2 versus m1	− 0.73480	− 0.22935	0.08239	− 0.92398	0.35550
m2/m2 versus m1/m1	− 0.28662	− 0.66670	0.09346	− 1.47802	0.13940
m1/m2 versus m1/m1	− 0.05268	− 0.27394	0.16858	− 0.46663	0.64077
m2/m2 + m1/m2 versus m1/m1	− 0.12988	− 0.28936	0.02960	− 1.59614	0.11046
m2/m2 versus m1/m1 + m1/m2	− 0.05904	− 0.33055	0.21247	− 0.42619	0.66997
**Ethnicity**					
m2 versus m1*	− 0.26779	− 0.45450	− 0.08108	− 2.81115	0.00494
m2/m2 versus m1/m1*	− 1.57249	− 2.72382	− 0.42115	− 2.67691	0.00743
m1/m2 versus m1/m1	− 0.19527	− 0.40773	0.01719	− 1.80137	0.07164
m2/m2 + m1/m2 versus m1/m1*	− 0.33727	− 0.44801	− 0.22654	− 5.96957	< 0.00001
m2/m2 versus m1/m1 + m1/m2*	− 1.74260	− 2.88511	− 0.60010	− 2.98943	0.00279

Sign of “*” in front of each genetic model means the correlation is statistically significant (*P* < 0.05). *CI* Confidence interval.

## Discussion

A recent systematic review reported that 242 genes have associated with the risk of HNC^74^. Our meta-analysis reported the association of one of the polymorphisms (*CYP1A1 MspI*) in these genes with the HNC susceptibility. The results were stable and showed elevated risks of m2 allele and m2/m2 and m1/m2 genotypes in HNC patients with enough samples that the results were under the influence of the ethnicity, the tumor type, and the control source. In addition, the sample size, the tumor type, and the ethnicity could be confounding factors on the results.

A 5.71-fold risk of nasopharyngeal cancer has been reported in cases carrying glutathione-S-transferases (*GSTs*) such as *GSTT1*, *GSTM1*, and *CYP1A1 MspI* genotypes, suggesting that cross-linking between these genes may modulate nasopharyngeal cancer susceptibility, with similar results reported in HNCs^17,46,62,71,75^. As the results of one study showed, *CYP1A1* polymorphisms alone were not related to an increased risk of oral cancer and the moderate risk for oral cancer was combining this polymorphism with *GST* polymorphisms^69^. Cha et al.^76^ showed the role of combined genotypes of *CYP1A1* m2/m2 and *GSTM1* null in the oral cancer risk. *Cyp1A1 MspI* polymorphism in lung cancer was associated with PAH-DNA adduct levels^77^ and the frequency of p53 gene mutations^78^. Smokers had the significant elevated risk (OR 7.13, *P* < 0.0001) of nasopharyngeal cancer among individuals carrying *CYP1A1 MspI* m2/m2 + m1/m2 genotype^71^.

One study in Northeast India found that the susceptibility to HNC related to tobacco and alcohol consumption is modulated by *CYP1A1 MspI* polymorphism, showing the interaction of gene-environment in prediction the HNC susceptibility and therefore this polymorphism is a predisposing risk factor for HNC^70^. In addition, another study reported tobacco use (particularly tobacco chewing) appeared as a significant moderator in cases with variant genotypes of *CYP1A1* in India^69^. Sharma et al.^65^ expressed that *CYP1A1* gene haplotype (C2453A, A2455G, and T3801C) frequency distribution in HNC patients was significantly higher than controls. Therefore, it is important to consider the haplotype and the combined impacts of genetic and environmental factors when examining the genetic risk to complex illnesses such as HNC^62,65^.

The discrepancies between results of the association between *CYP1A1* polymorphisms and HNCs in Indians may be due to ethnic differences in culture, linguistics, and diets in this population, or they may be because of a difference in the sample size of the studies^63^. As our meta-analysis confirmed that the sample size was a confounding factor on the association and increasing the sample size, OR increased.

Wang et al.^79^ reported that the association between *CYP1A1* polymorphisms and the risk of HNC could be affected by tumor type as that they observe an elevated risk of laryngeal and pharyngeal cancers, but no for oral cancer. Another study^70^ showed that the m2/m2 genotype of *CYP1A1 MspI* polymorphism had a significantly elevated risk in oral cancer patients, but there was no significant relationship between this polymorphism and pharyngeal and laryngeal cancers that one review^80^ confirmed it. Also, Sam et al.^63^ showed the association between m1/m2 genotype had just significant risk in laryngeal and pharyngeal cancers, not oral cancer. In our meta-analysis, the m1/m2 genotype had just a significant association with pharyngeal cancer and m2/m2 just in oral and laryngeal cancers.

Seven meta-analyses^21–27^ reported the association between *CYP1A1 MspI* polymorphism and the risk of HNCs. Two meta-analyses^22,25^ illustrated that the *CYP1A1 Msp*I polymorphism may be associated with oral cancer susceptibility in Asians as well as Xie et al.^81^ in a stratified analysis by ethnicity, showed significant evidence of the association of *CYP1A1 MspI* polymorphism with the HNC risk in Asian ethnicity, but not mixed and Caucasian ethnicities. These results showed the impact of ethnicity on the relationship between *CYP1A1 MspI* polymorphism and the HNC risk as the meta-analysis of He et al.^24^ and our meta-analysis reported. In addition, our meta-analysis showed an association between other cancers (laryngeal and pharyngeal cancers), both in Asians and in other ethnicities (Caucasian and mixed ethnicities). Some studies^82–84^ and our meta-analysis to follow them, classified Indians in Caucasian ethnicity and some other studies^85,86^ as Asians, but based other studies^87,88^, Indians include several ethnicities (mixed). One possible difference between the results of studies can be due to the different classification of the ethnicity for each region. Therefore, it should be noted that there is a need for a comprehensive classification to select the type of ethnicity of each country or region in the future so that the results of meta-analyzes based on the ethnicity be more homogeneous. In addition, in the meta-analyzes mentioned^21–27^, the number of studies was different and this could be another reason for the difference between their results. So, more studies are needed in different areas in the world to reduce this difference in results between studies. As the results of different studies and their contradictions showed, the relationship between this polymorphism and HNC risk is influenced by various factors, and paying attention to the effective factors in future studies can provide a way to find more dominant factors. As a result, treatment of these patients and as a result of increasing their survival can be done more easily and under more effective and better conditions.

Apart from several strengths (enough samples, stability of the results, and lack of publication bias across the studies), the present meta-analysis also had some limitations as (1) High heterogeneity between the studies. (2) Few numbers of studies in Asian and mixed ethnicities. (3) The impact of risk factors on the results with different distributions in included studies. (4) We just included published studies.

### Conclusions

The findings of the present meta-analysis recommended the association between *CYP1A1 MspI* polymorphism and the HNC susceptibility with enough samples and stable results. The ethnicity, the tumor type, the control source, and the sample size were significant risk factors for the results. Therefore, pay attention to these factors can be important in relation to the association of *CYP1A1 MspI* polymorphism and the HNC risk in future studies. In addition, well-designed studies with large samples in various areas of the world with precise matching criteria are required to reveal the present meta-analysis conclusions.

## Data Availability

The datasets used and/or analyzed during the current study are available from the corresponding author on reasonable request.

## Abbreviations

HNC: Head and neck cancer
CYP: Cytochrome P450
OR: Odds ratio
CI: Confidence interval
PAH: Polycyclic aromatic hydrocarbon
TSA: Trial sequential analysis
GST: Glutathione-S-transferase
